# Facilitation of Visual Perception in Head Direction: Visual Attention Modulation Based on Head Direction

**DOI:** 10.1371/journal.pone.0124367

**Published:** 2015-04-28

**Authors:** Ryoichi Nakashima, Satoshi Shioiri

**Affiliations:** Tohoku University, and Japan Science and Technology Agency, Crest, Sendai, Japan; University of Texas at Dallas, UNITED STATES

## Abstract

People usually see things using frontal viewing, and avoid lateral viewing (or eccentric gaze) where the directions of the head and eyes are largely different. Lateral viewing interferes with attentive visual search performance, probably because the head is directed away from the target and/or because the head and eyes are misaligned. In this study, we examined which of these factors is the primary one for interference by conducting a visual identification experiment where a target was presented in the peripheral visual field. The critical manipulation was the participants’ head direction and fixation position: the head was directed to the fixation location, the target position, or the opposite side of the fixation. The performance was highest when the head was directed to the target position even when there was misalignment of the head and eye, suggesting that visual perception can be influenced by both head direction and fixation position.

## Introduction

Generally, people look at objects in one local area at a time and shift their gaze in series to scan and comprehend the surrounding visual environment, because they cannot and do not process all the information simultaneously. When performing everyday visual tasks, saccadic eye movements usually occur over a range of 30° or less, e.g., [1], [2], [3], although this can extend to more than 100° at maximum. When shifting the gaze to an object far from the current fixation point, people usually move both their eyes and head to face the object in a coordinated manner, e.g., [4], [5], [6], [7], [8], [9]. For example, when people detect another person’s face, they usually direct their head to the face in order to gaze at it [4]. These facts suggest that people avoid lateral viewing, where the directions of the head and eyes are largely different (i.e., eccentric gaze).

Previous studies examining eye-head coupling during saccades [5], [6], [7], [8], [9], [10] have indicated that head movement during a gaze shift depends on a cost/benefit judgment. It is more difficult to move the head than the eyes because of its greater weight, which represents the cost of head movement. However, fixation accuracy and stability decrease at far-eccentric eye positions because of the constraint imposed by the ocular muscles [10], which represents the relative benefit of head movement (or alternatively, the cost of no head movement).

Few studies to date have examined the relationship between head direction/movement and visual perception, e.g., [11], and little is known about how lateral viewing influences visual perception, despite its possible importance. To our knowledge, only our recent study has directly examined the effect of lateral viewing on visual perception [12]. This study compared preattentive and attentive visual search performances between conditions where participants directed both their eyes and head to visual stimuli (i.e., frontal viewing, or looking at straight ahead) and where they directed only their eyes to visual stimuli (i.e., lateral viewing, or looking in a direction different from head direction) and found that attentive visual search performance was poorer in lateral viewing. There are two possible explanations for the observed decline in lateral viewing performance (or better frontal viewing performance). First, the directions of the head and eyes differed in lateral viewing, that is, they were misaligned (and the directions of the head and eyes were the same in frontal viewing, i.e., they were aligned). Second, the head was not directed toward the position of the stimulus in lateral viewing (and it was in frontal viewing).

In this study, we examined which of these two factors causes the difference in attentive visual search performance between the lateral and frontal viewing conditions. For this purpose, we manipulated the head direction of participants relative to the eye direction and also relative to a target presented in their peripheral visual field.

## Experiment 1

We conducted a visual identification task where visual stimuli were presented in participants’ peripheral vision. One target stimulus, a T-shaped figure, and either of two distractor stimuli, L- and O-shaped figures, were prepared. During the task, the target figure and one of the distractors were presented. We used the distractor conditions to manipulate the task difficulty, as discriminating “T” from “O” was assumed to be easier than from “L”, cf. [12], [13]. To eliminate possible eye movements during visual search, the stimuli were presented briefly and the accuracy was measured as the dependent measure. Generally, correct response measurements (i.e., accuracy) under limited presentation durations usually provide complimentary results to RT measures in visual search, e.g., [14], [15], [16].

**Fig 1:**
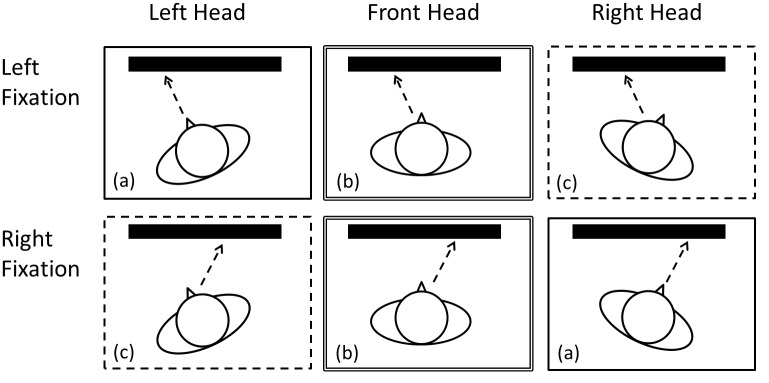
Experimental setup. Based on the relationship between head (and body) direction and the fixation position, three experimental conditions were defined: (a) head directed to the fixation cross (figures surrounded by a solid line), (b) head directed to the stimulus position (figures surrounded by a double line), and (c) head directed to the opposite side of the fixation from the stimulus (figures surrounded by a dashed line). Dashed arrow indicates eye direction in each figure.

The target and distractor stimuli were always presented at the center of the display. The fixation cross, which participants were instructed to gaze at throughout the trial, was presented to the left or right of the display center. The participants’ head direction was manipulated to the left, front, or right. In the left (or right) head direction condition, the head was directed to the left (or right) fixation position. In the front head direction condition, the head was directed to the stimulus position. The head and body directions were always kept the same in this study. Combining the fixation position and the head direction, we defined three conditions, where the head was directed to (a) the fixation position, (b) the stimulus position, or (c) the position opposite of the fixation (Fig 1). Our main interest in this study was to compare the performances between conditions where the head was directed to (a) the fixation cross (i.e., directions of the eyes and head aligned) and (b) the stimulus.

### Method

#### Participants

Participants in Experiment 1 were 12 undergraduate or graduate students (age: 22–24 years). All participants had normal or corrected-to-normal vision with contact lenses and were naïve to the purpose of this study. None had any problems looking at the stimuli via lateral viewing. All experiments were approved by the institutional review board of Tohoku University, and written informed consent was obtained from all participants. Based on the results of our previous study [12], we assumed that the effect size of the head direction on visual perception should be large (η_p_^2^ = .20 for the main effect of head direction in Experiment 1). A power analysis using G*power 3.1 [17] indicated that this number of participants allowed for examination of the effect of head direction, our main interest in this study, at a power > 80% to test large effect size (although the calculated f was 0.5, we conservatively used f = 0.4; see [18]) with a Type 1 error (α < .05).

#### Apparatus

MATLAB software and the Psychophysics Toolbox [19], [20] were used for stimulus presentation and response recordings. A 37-inch liquid crystal display (1280 × 720 pixels) was used for stimulus display.

#### Stimuli

The target stimulus was a white T-shaped figure (325.6 cd/m^2^) of 1.5° × 1.5° of visual angle at a viewing distance of 60 cm. It was rotated 90° to the left or right of vertical. The distractor stimulus was a white L-shaped or O-shaped figure (325.6 cd/m^2^) of 1.5° × 1.5°. The L-shaped figure was rotated 0°, 90°, 180°, or 270°. The stimuli were presented on a gray background (63.8 cd/m^2^). The mask stimulus was a white 8-shaped figure (325.6 cd/m^2^) of 1.5° × 1.5°. We used stimuli larger than those in our previous study [12], because they were presented in the peripheral vision, where visual acuity is low, e.g., [21], [22], [23], [24]. Stimulus size was determined to enable the participants to discriminate the target direction at the eccentric stimulus presentation, e.g., [25], [26], see also [27].

The stimulus display consisted of the target (T) and one of the distractors (L or O) arranged side-by-side (T/L task and T/O task). Target location (left or right) was randomly determined each trial. A digital number 8 was presented to mask each stimulus figure after its presentation.

#### Procedure

**Fig 2:**
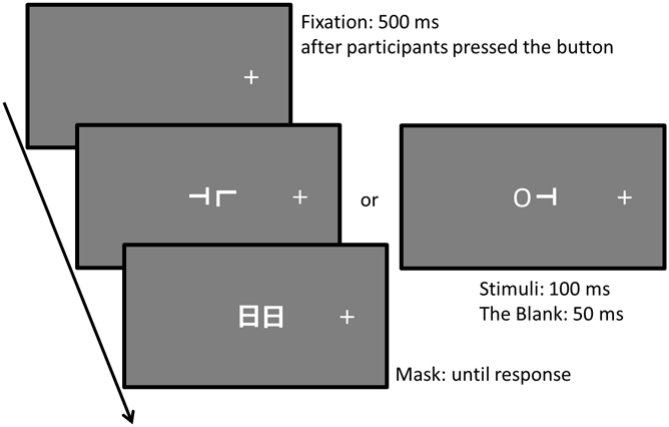
The sequence of an experimental trial in Experiment 1. Examples of trials where the fixation position was on the right of the display. In Experiment 1, fixation was randomly presented on the right or left of the display in each block. In this figure, the correct response was “Left” in both stimulus displays.

All participants viewed the stimulus with both eyes, because our previous study [12] showed no significant difference between the performances of two-eye viewing and one-eye viewing. At the beginning of a trial, the fixation cross was presented at 15° to the left or right from the center of the display (see Fig 2). Participants were instructed to fixate on the cross and press a button to start the trial. Five hundred milliseconds later, a target distractor pair was presented at the center of the display for 100 ms, followed by a blank display (50 ms) and then a mask display (until response). The task was to identify the direction of the target (i.e., whether the bottom of the T was pointed to the right or left) as accurately as possible, without time limitation. Participants were told to gaze at the fixation cross during a trial, but were not required to maintain this between trials to minimize possible fatigue from lateral viewing.

Head direction (front, left, or right) was fixed by a forehead and chin rest throughout an experimental block. To change the head direction condition, we rotated the chin and forehead rest between the experimental blocks. In the front condition, the head was directed to the center of the display, while in the left (right) condition, it was directed 15° to the left (right) of center. As described above, we defined three conditions: (a) head directed at the fixation cross (i.e., 0° difference between eye and head directions), (b) head directed to the stimulus position, (i.e., 15° difference), and (c) head directed to the opposite side of the fixation from the stimulus, (i.e., 30° difference).

The three head direction conditions were blocked, and the block order was randomized across participants. Each block consisted of 320 trials: 80 trials in each of the four conditions created by a 2 (fixation position: left or right) × 2 (task: T/L or T/O) factorial design. The trial order was randomized in each block. We compared accuracies (i.e., the percentages of correct responses) among these conditions.

### Results

**Fig 3:**
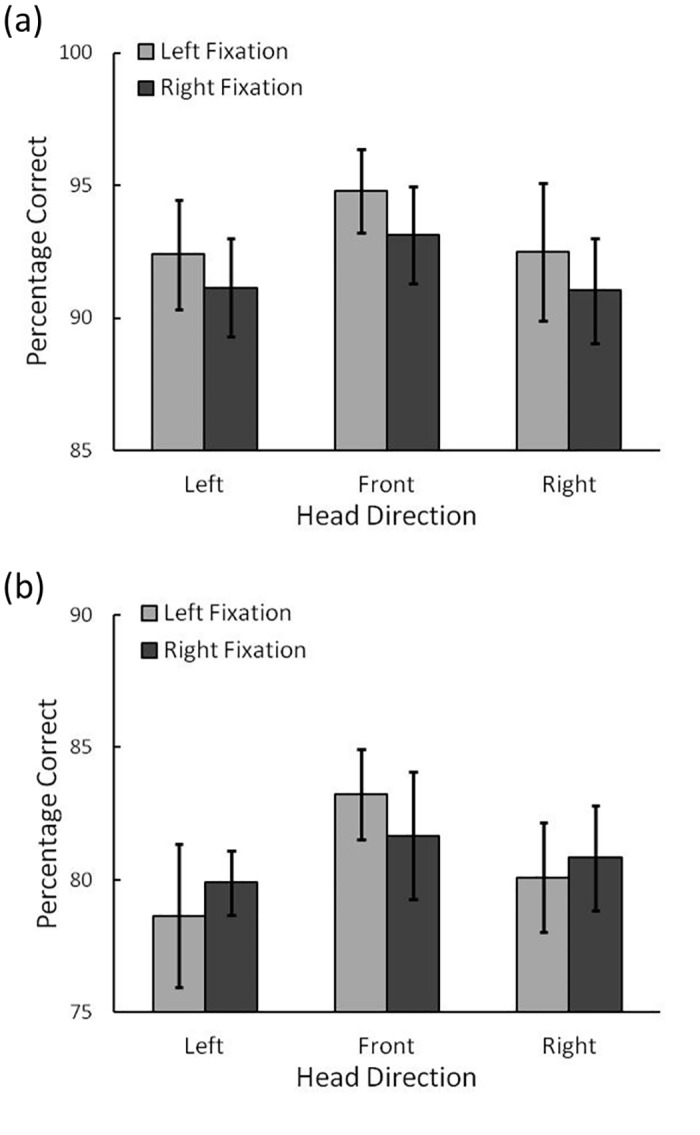
Results of Experiment 1. The percentage of correct responses for the (a) T/O task (low cognitive load) and (b) T/L task (high cognitive load) in Experiment 1. Error bars indicate 95% confidence intervals.

As described above, reaction time (RT) for the identification of the target was not very informative in the present experiments, because the duration of stimulus presentation was limited and response speed was not emphasized. Thus, we mainly analyzed participant accuracy, that is, the percentage of correct responses among the conditions (see Fig 3). We conducted an analysis of variance (ANOVA) on the performance with factors for task (T/L vs. T/O), head direction (left vs. front vs. right), and fixation position (left vs. right) as within-participants factors. The main effect of task was significant, indicating that performance was higher in the T/O than in the T/L task, *F*(1, 11) = 80.40, *p* < .001, η_p_^2^ = .88. The main effect of head direction was also significant, *F*(2, 22) = 9.73, *p* < .01, η_p_^2^ = .47. Performance in the head front condition was higher than in the head left and right conditions, *p*s < .001. The difference in performances between the head left and right conditions was not significant, *p* = .39. The main effect of fixation position and the interactions did not reach significance, fixation position: *F*(1, 11) = .37, *p* = .55, head × fixation: *F*(2, 22) = .75, *p* = .48, head × task: *F*(2, 22) = .27, *p* = .76, task × fixation: *F*(2, 22) = .91, *p* = .36, three-way interaction: *F*(2, 22) = .42, *p* = .66.

We analyzed RT of the correct response trials to examine whether there was evidence of speed-accuracy tradeoff. Although our experiment was not designed to use RT as a measure of the performance (no speeded responses were required), it was possible that some participants tried to respond quickly with less consideration of accuracy in some conditions. An ANOVA revealed that only the main effect of task was significant, indicating that the response was faster in the T/O than in the T/L task, *F*(1, 11) = 54.02, *p* < .001, η_p_^2^ = .83. In the T/O task, the mean RT was 597 ms when averaged across all conditions and participants and it was 717 ms in the T/L task (see S1 Table for details). No other main effects or interactions were significant, head direction: *F*(2, 22) = 1.14, *p* = .33, fixation position: *F*(1, 11) = 1.72, *p* = .22, head × fixation: *F*(2, 22) = 1.02, *p* = .38, head × task: *F*(2, 22) = 1.45, *p* = .25, task × fixation: *F*(2, 22) = .11, *p* = .74, three-way interaction: *F*(2, 22) = 1.29, *p* = .29. The results suggest that speed-accuracy tradeoff was not an issue.

The supporting information file (S1 Table) shows percentage of correct responses and RT data for each condition for each participant and experiment.

### Discussion

Visual identification was best when the head was directed toward the target stimulus, even if the eyes were fixed away from the target. Accurate visual identification in this task required participants to orient covert attention, e.g., [28], [29] to the location of the stimulus. Thus, better performance when the head was directed to the target stimulus suggests that attention is biased toward the direction of the head rather than the eyes.

Better performance in the T/O task than in the T/L task confirmed the successful manipulation of task difficulty, cf. [12], [13]. Furthermore, the lack of any significant interaction between task and head direction suggested that the effect of the head direction on visual processing was independent of task difficulty.

In this task, whether the directions of the head and eyes were aligned (e.g., left fixation and left head) or misaligned (e.g., left fixation and right head) had little or no effect on visual processing. In these conditions, the retinal stimulations were the same. Therefore, the lateral viewing itself, due to misalignment of the head and eyes by 30°, was not expected to interfere with visual perception.

The results confirm our previous finding that head direction influences visual perception. The present results, however, are apparently inconsistent with the finding that head direction influences visual performance only when attention is required to perform the task, cf. [30], [31], [32], [33], [34]. In a typical visual search experiment, such as in our previous study, a search for “T” among “O”s is characterized as a parallel search, and performance is independent of the number of items, whereas a search for “T” among “L”s is characterized as a serial search, and performance depends on the number of items [12], [13]. The previous study found a significant effect of head direction on visual performance for the T/L task but not for the T/O task. In contrast, similar head direction effects were found in the present results for both T/O and T/L tasks. However, the present experiment used peripherally presented stimuli, and the difference between parallel and serial searches was ambiguous for peripheral vision. In peripheral vision, a serial search property is found in some experimental conditions where parallel search property is found in the central vision [35]. Both T/L and T/O searches might be processed using a common underlying mechanism in this experiment.

## Experiment 2

Before we conclude that visual processing is facilitated by the head direction and that lateral viewing by 30° itself has no influence on visual perception, we should consider two issues. First, we should examine the long-term effect of lateral viewing on visual perception. If there is a long-term effect of lateral viewing, such as from extraocular muscle tension, lateral viewing may influence all later trials within a block similarly. In Experiment 1, frontal viewing and lateral viewing were manipulated within a block by varying the fixation position from trial to trial. This manipulation may reduce the difference between the two types of viewing if the long-term effect of lateral viewing spreads across trials in a block, consequently showing no difference between performance in frontal viewing and lateral viewing. To examine this effect specifically, the head direction and the fixation position conditions were blocked. That is, the eye direction relative to the head direction was kept the same throughout each block.

Second, fixation tends to occur in the direction the head is facing (central fixation bias; e.g., [37], [38], [39]). Accidental eye movements to the stimulus in the head front condition, therefore, might have led to better performance. To eliminate this possibility, we added filler trials where the stimulus was presented at the fixation position. In the filler trial, participants looked at the stimulus with the central vision and the task was very easy. Fixating the stimulus at the center of the display, rather than at the fixation point, would affect performance on the filler trials.

### Method

#### Participants

Participants in Experiment 2 were 13 undergraduate or graduate students (age: 22–31 years), 4 of whom had participated in Experiment 1. All participants had normal or corrected-to-normal vision and were naïve to the purpose of the study.

#### Apparatus and stimuli

Apparatus and stimuli were the same as those in Experiment 1, except only the L-shaped figure was used as a distractor because the effect of the head direction was similar between the two tasks in Experiment 1.

#### Procedure

**Fig 4:**
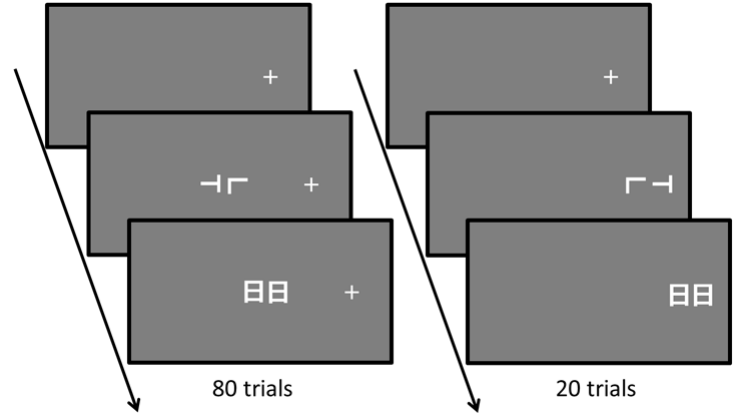
The sequence of an experimental trial in Experiment 2. Examples of trials where the fixation position was on the right of the display. There were 80 trials where the stimulus was presented at the center of the display (left) and 20 trials where the stimulus was presented at the location of the fixation cross (right) in each block. In this figure, the correct response was “Left” in both trials.

The procedure was the same as that of Experiment 1, except for the following three manipulations. First, only the T/L task was conducted. Second, the head direction and the fixation position conditions were blocked into six blocks (3 head directions × 2 fixation positions). Third, in each block, there were 80 trials where the stimulus was presented at the center of the display and 20 trials where it was presented at the location of the fixation cross (Fig 4). The block order was randomized across participants. The trial order was randomized in each block.

### Results and Discussion

The percentage of correct responses when the stimulus was presented at the fixation position was very high (left fixation condition: 98.8 ± .8% in head left, 98.8 ± .6% in head front, and 99.2 ± .5% in head right conditions; right fixation condition: 99.2 ± .5%, 99.2 ± .5%, and 98.8 ± .6%, respectively; mean ± SE), with no significant differences among conditions, head: *F*(2, 24) = 0, *p* = 1, fixation: *F*(1, 12) = .07, *p* = .79, head × fixation: head: *F*(2, 24) = .23, *p* = .79. These results confirm the occurrence of eye fixation at the indicated location for at least 98% of trials. This indicates that effect of eye movements, if any, should be about 1% or less of trials because the error rates include mistakes of key presses. Mean RTs were about 550 ms (see S1 Table).

**Fig 5:**
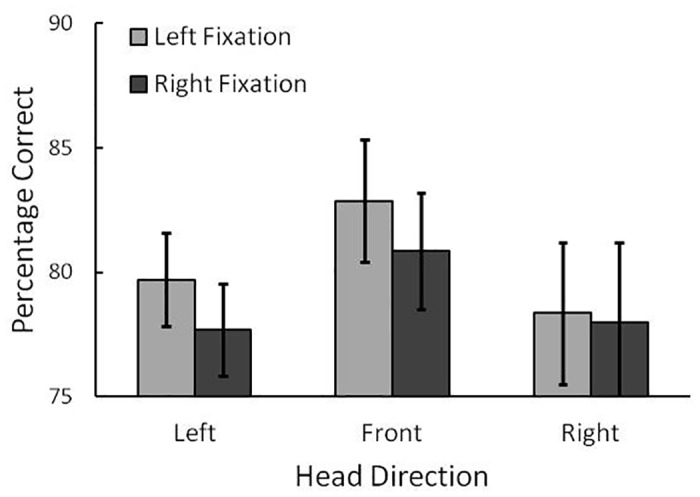
Results of Experiment 2. The percentage of correct responses for the task where the stimulus was presented at the center of the display in Experiment 2. Error bars indicate 95% confidence intervals.

Fig 5 shows the percentage of correct responses in each condition in the main task. The results were very similar to those of Experiment 1. An ANOVA revealed a main effect of head direction, *F*(1, 12) = 4.34, *p* = .02, η_p_^2^ = .27. Performance in the head front condition was better than in the head left and right conditions, *p*s < .02. The difference in performance between the head left and right conditions was not significant, *p* = .70. The main effect of fixation position and the interactions did not reach significance, fixation: *F*(1, 12) = .52, *p* = .48, head × fixation: head: *F*(2, 24) = .30, *p* = .74.

The mean RT was 778 ms when averaged across all conditions and participants (see S1 Table for details). No main effects or interactions were significant, head direction: *F*(2, 24) = .64, *p* = .53, fixation position: *F*(1, 12) = .25, *p* = .62, head × fixation: *F*(2, 24) = .95, *p* = .39. RT results suggest no speed-accuracy tradeoff in this experiment.

Even when the participants clearly gazed at the fixation cross, performance was highest in the head front condition. This result confirms that visual processing is facilitated by head direction and suggests that whether the directions of head and eyes are aligned (e.g., left head and left fixation) or misaligned (e.g., right head and left fixation) has little or no effect on visual processing.

## General Discussion

Whether the head moves or not to look at an object may be based on an evaluation of the cost/benefit in the controlling process of head and eye movements, e.g., [5], [6], [7], [8], [9] on one hand. Eye position in the head may also impact visual perception on the other hand. Our previous study found that lateral viewing, where the directions of the eyes and head are largely misaligned, interferes with attentive visual search performance [12]. In this study, we examined the relationship between the head direction and the eye direction in head and visual perception and found that head direction, but not eye direction in head, modulates visual perception. For identical retinal stimulation, performance was higher when the head was directed to the stimulus than when it was directed to other locations. We also showed that lateral viewing by 30° itself (i.e., the misalignment of both the head and eyes by 30°) did not interfere with visual perception in the present experiments. Therefore, the effect of lateral viewing on visual perception cannot be attributed to eye and head misalignment. Rather, the tendency of visual attention to be oriented to the head direction may be the primary factor in the lateral viewing effect. We conclude that visual performance is facilitated when the head is directed to the stimulus and/or deteriorates when the head is directed to other locations. Some previous studies reported that attention shift influences the head and eye movements, e.g., [40], [41]. This study suggests the converse effect that the head direction can influence attention shift.

Why, then, does visual attention tend to follow head direction, provided that attention is (partially) focused on the head direction? One possible explanation is the consequence of attention bias toward the goal of eye movements. People usually see things straight ahead, with the eyes following the head direction (the central fixation bias; [36]). When eyes direct to the lateral position, eye movements toward the center of the head are faster and shorter in latency than those toward the periphery of the head, e.g., [42], [43], [44]. Since attention moves to the saccade goal prior to the eye movements, e.g., [45], [46], [47], [48], [49], attention also tends to move to the head direction when the eyes direct to the lateral position. Attention shifts to the saccade goal not only for voluntary eye movement, but also for involuntary eye movement [50], where the eyes direct downward involuntarily during an eye blink and attention moves downward before the eye blink. In sum, the eyes may tend to involuntarily direct to the head direction, and accordingly attention tends to be biased toward the head direction.

In conclusion, we found that head direction is a factor modulating visual perception. The effect, at least in part, might be caused by attention allocation in the direction the head is facing. This is distinct from the coordination in motor control of the eye and head that has been extensively investigated in the literature, e.g., [5], [6], [7], [8], [9]. The present experiments show that head movements alone toward a stimulus can improve visual processing. We suggest that eye and head direction are not only controlled through the coordination of motor processes, but they also are controlled through the coordination of perceptual processes.

## Supporting Information

S1 Table Data of the percentage of correct responses and RT for each condition for each participant in Experiments 1 and 2. (XLSX)
